# Synthesis of diketopyrrolopyrrole-based polymers with polydimethylsiloxane side chains and their application in organic field-effect transistors

**DOI:** 10.1098/rsos.172025

**Published:** 2018-03-14

**Authors:** Inori Ohnishi, Kazuhito Hashimoto, Keisuke Tajima

**Affiliations:** 1 RIKEN Center for Emergent Matter Science (CEMS), 2-1 Hirosawa, Wako 351-0198, Japan; 2 Department of Applied Chemistry, Graduate School of Engineering, The University of Tokyo, 7-3-1 Hongo, Bunkyo-ku, Tokyo 113-8656, Japan

**Keywords:** semiconducting polymer, solubility, organic field-effect transistor, crystallinity, thin film

## Abstract

Linear polydimethylsiloxane (PDMS) was investigated as a solubilizing group for π-conjugated polymers with the aim of combining high solubility in organic solvents with the molecular packing in solid films that is advantageous for charge transport. Diketopyrrolopyrrole-based copolymers with different contents and substitution patterns of the PDMS side chains were synthesized and evaluated for application in organic field-effect transistors. The PDMS side chains greatly increased the solubility of the polymers and led to shorter *d*-spacings of the π-stacking in the thin films compared with polymers containing conventional branched alkyl side chains.

## 1. Introduction

Organic semiconducting polymers have been widely studied for use in organic electronic devices such as organic field-effect transistors (OFETs), organic light-emitting diodes and organic photovoltaics. An important feature of this class of materials is their capacity to be solution processed into thin films through continuous coating or printing processes. This permits large-area and low-cost fabrication of the devices, which is advantageous compared with the conventional methods based on inorganic semiconductors. Many polymers with various π-conjugated main chains have been developed to achieve high-performance characteristics, such as efficient light absorption and emission, and high charge mobility in the optoelectronic devices [1–5]. In general, larger π-systems are preferred for realizing superior electronic properties, because the delocalization of the charges and the strong intermolecular interactions can lead to high charge mobility in the thin films. These strong intermolecular interactions, however, often cause poor solubility of the polymers in organic solvents. The introduction of linear alkyl chains improves the solubility to some extent, but such polymers tend to crystallize in the solid state when the chains contain more than five carbons [6]. To avoid this, long and branched alkyl solubilizing groups are often introduced into the π-conjugated polymers as the side chains. Although this can greatly improve the solubility, the large steric hindrance due to the bulky side chains impairs the π–π interaction between the main chains and leads to reduced charge mobility. Optimizing this trade-off relationship is a key target in the development of new π-conjugated polymers.

It has been reported that moving the branching point of the alkyl side chains away from the main chain can improve the solubility of the polymers while maintaining their electronic properties [7–13]. Several alternative solubilizing side chains besides branched alkyl chains have also been developed [14,15]. Mei *et al*. [16,17] demonstrated that the introduction of a linear alkyl side chain terminated with a branched trisiloxane into isoindigo-based conjugated polymers can afford high solubilities similar to those obtained using long-branched alkyl chains. As a result of the lower steric hindrance of the side chains, the polymers exhibited a smaller π-stacking distance in the films and higher charge mobility. Meng *et al*. [18] reported that the introduction of oligo(ethylene glycol) instead of linear alkyl chains into a fluorene-based alternating copolymer led to a decrease in the π–π stacking distance in the film state without impairing the solubility. The exploration of new solubilizing side chains that can simultaneously afford good solubility in solution and high electric performance in thin films is therefore of general importance.

**Figure 1.**
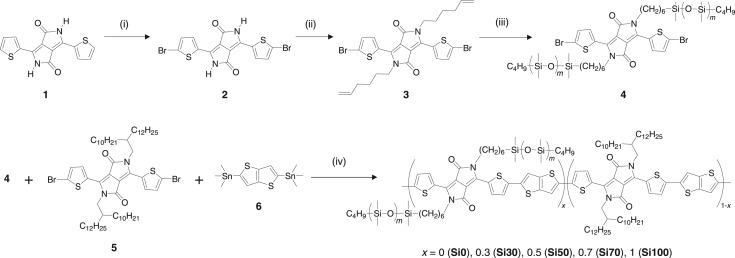
Synthetic routes to the DPP monomer with PDMS side chains and to the random copolymers with various PDMS contents. (i) NBS, CHCl_3_; (ii) 6-bromo-1-hexene, K_2_CO_3_, DMF; (iii) monohydride-terminated PDMS, Karstedt's catalyst, toluene; (iv) Pd_2_(dba)_3_·CHCl_3_, P(*o*-tolyl)_3_.

In this report, a linear polydimethylsiloxane (PDMS) was used as the solubilizing side chain in semiconducting polymers. As Si–O–Si possesses a greater bond length and a larger angle than CH_2_–CH_2_–CH_2_, the torsional potential is lower and therefore the PDMS chains are much more flexible than the alkyl chains [19]. This may lead to better solubility of the resulting polymers than the use of alkyl chains without disturbing the packing of the main chain. We synthesized diketopyrrolopyrrole (DPP) units with linear PDMS as the side chain and copolymerized them with thieno[3,2-*b*]thiophene (figure 1) [20–22]. The main-chain structure of this alternating copolymer has been reported to afford good ambipolar transport in OFETs [23–27], but these polymers are soluble at room temperature only with long-branched alkyl chains as a result of the strong intermolecular interactions.

## 2. Results

The DPP monomer unit with the PDMS side chains was synthesized as shown in figure 1. The thiophene-bearing DPP derivative **1** was first brominated at the 5-position of the thiophenes to afford **2**, which was subsequently *N*-alkylated with 6-bromo-1-hexene under basic conditions to give **3**. PDMS terminated with hydride and butyl groups (*M*_w_ = 800–900, degree of polymerization = 9.2–10.6) was attached to the alkyl ends via a hydrosilylation reaction in the presence of Karstedt's catalyst to afford **4** [28]. It should be noted that when we conducted the bromination step after the *N*-alkylation or the hydrosilylation, the reaction was complicated by overbromination at the side chains or difficulties in the purification, respectively. The monomer **4** was alternatingly copolymerized with 2,5-bis(trimethylstannyl)thieno[3,2-*b*]thiophene (**6**) in combination with the DPP monomer containing 2-decyltetradecyl side chains (**5**), as shown in figure 1. While the molar feed ratio between the sum of DPP monomers and **6** was fixed at 1 : 1, the feed ratio of **4** to **5** was changed from 0% (i.e. the polymer with only branched alkyl side chains) to 30%, 50%, 70% and 100% (i.e. the polymer with only PDMS side chains). The resulting polymers are hereafter referred to as Si0, Si30, Si50, Si70 and Si100, respectively.

The actual ratios of the PDMS side chain in the copolymers were estimated by elemental analysis (C, H and N) based on the molecular formulae and the assumption that the degree of polymerization (*m*) of PDMS is 10 (table 1). The observed ratios were close to the monomer feed ratios, suggesting that the monomers **4** and **5** were incorporated randomly into the main chain according to their feed ratios. The molecular weights of the polymers were higher than 100 kg mol^−1^, with the exception of Si100, which exhibited a significantly lower molecular weight and polydispersity index (PDI) than did the others. The reason for this low degree of polymerization is unclear, but it is plausible that the PDMS side chains of the monomer **4** start to cover the reactive end groups and hinder the access of the monomer **6** to the ends when the feed ratio becomes too high. It would be difficult to generalize the effects of the molecular weight on the charge mobility in OFET devices, but the previous report suggested that there could be a threshold molecular weight above which the crystallinity and the mobility become less sensitive to the molecular weight [29]. In the current case, *M*_n_ values of the polymers are larger than 100 kg mol^−1^, except for Si100, and as the absorption spectra were not sensitive to the molecular weights in this range, we expect that the electric properties of Si0, Si30, Si50 and Si70 are less dependent on the molecular weights.

**Table 1.** Summary of the properties of the polymers. PDI, polydispersity index.

polymer	Si0	Si30	Si50	Si70	Si100
feed ratio of **4**	0%	30%	50%	70%	100%
observed ratio of **4**^a^	—	27%	46%	68%	107%
*M*_n_ (kg mol^−1^)	111	228	176	115	39
PDI	3.6	8.4	4.0	3.4	1.3
solubility in CHCl_3_ (mg ml^−1^)	11	11	16	27	30

^a^Calculated from elemental analysis of the polymers based on an assumed degree of polymerization (*m*) of PDMS of 10.

As the ratio of the PDMS unit increased, the solubility of the polymers in CHCl_3_ at room temperature greatly increased from 11 mg ml^−1^ for Si0 to 27 mg ml^−1^ for Si70, even though the molecular weights were similar. Si100 showed an even higher solubility of 30 mg ml^−1^, although the much lower molecular weight may have contributed to this improvement. We also observed that Si70 and Si100 were soluble in CH_2_Cl_2_, while Si0, Si30 and Si50 were barely soluble in CH_2_Cl_2_. On the other hand, Si0 has a lower solubility in toluene and the solution undergoes gelation during the polymerization, so the molecular weight of Si0 can be limited by the solubility of the product. This may be a possible reason why the highest molecular weight was observed when the moderate amounts of PDMS side chain were introduced, where the solubility of the polymer and the reactivity of the monomer are balanced. These results indicate that the introduction of PDMS side chains improved the solubility of the copolymers.

**Figure 2.**
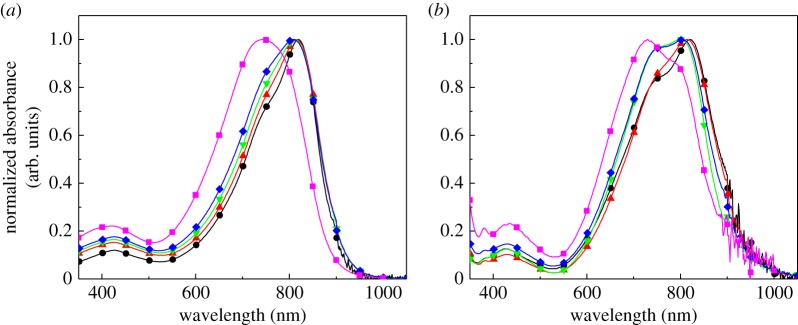
Normalized UV–vis absorption spectra of the copolymers in (*a*) CHCl_3_ solutions and (*b*) thin films. Circles, Si0; triangles, Si30; inverted triangles, Si50; diamonds, Si70; squares, Si100.

Figure 2 shows the UV–vis absorption spectra of the polymers in CHCl_3_ solution and in thin films. The solution spectra of the polymers were similar to each other with absorption maxima at approximately 810 nm, with the exception of Si100 which exhibited its absorption maximum at the shorter wavelength of 742 nm. This may be attributable to the lower molecular weight of Si100, which resulted in a shorter conjugation length than in the other polymers. In the thin-film spectra, the absorption bands of the polymers showed slight blue shifts relative to the solution spectra with more pronounced absorption shoulders at approximately 750 nm for Si0, Si30, Si50 and Si70. This enlargement of the vibronic structure has been observed previously for DPP-based polymers, indicating the presence of interchain interactions in the solid state [21]. This implies that the PDMS side chain did not prevent the interactions between the main chains. Interestingly, in the thin films, the absorption shoulders were more intense for Si50 and Si70 than for Si0 and Si30. This suggests that increasing the proportion of PDMS side chains may facilitate the aggregation of the main chains in the films. The PDMS side chains are longer than the branched alkyl chains, but are also linear and more flexible. Therefore, it is reasonable to expect less steric hindrance than with the branched alkyl chains and better packing of the main chains. The ionization potentials measured by photoelectron spectroscopy in air were 5.23 eV, 5.27 eV, 5.25 eV, 5.20 eV and 5.26 eV for Si0, Si30, Si50, Si70 and Si100, respectively, indicating that the electronic energy levels are similar among the copolymers.

**Figure 3.**
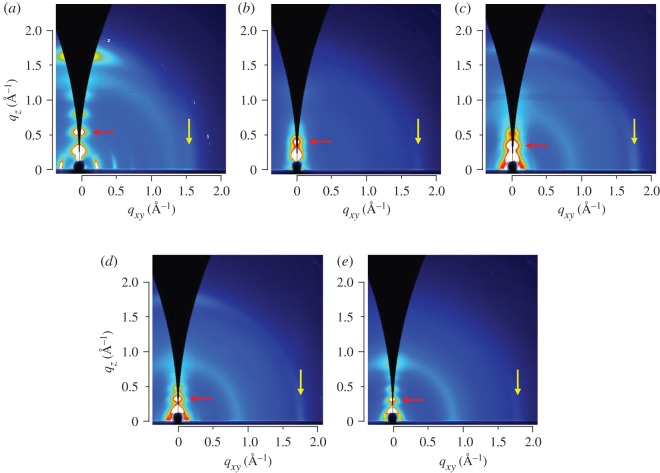
2D GIWAXS patterns of (*a*) Si0, (*b*) Si30, (*c*) Si50, (*d*) Si70 and (*e*) Si100 films. The red and yellow arrows indicate the second-order lamellar and π-stacking diffractions, respectively. The samples were annealed at 250°C for 5 min.

To further analyse the structure in the thin films, both grazing-incidence wide-angle X-ray scattering (GIWAXS) and X-ray diffraction (XRD) with a goniometer were performed on the thin-film samples. Figure 3 shows the two-dimensional (2D) GIWAXS patterns of the polymers after annealing at 250°C for 5 min. The Si0, Si50 and Si70 films exhibited a mixture of face-on and edge-on orientations, whereas the Si30 and Si100 films possessed a mostly edge-on orientation. As the content of PDMS in the copolymers increased, the amorphous halo observed at *q* = 0.9 Å^−1^ became more obvious, which coincides with the increase of the amorphous PDMS domains. At the same time, the peaks in the out-of-plane direction moved to smaller angles (red arrows), indicating the increase of the lamellar distance. By contrast, the peaks in the in-plane direction shifted to larger angles (yellow arrows), indicating the decrease of the π-stacking distance. Table 2 summarizes the lamellar and π-stacking distances obtained from the out-of-plane (*θ*/2*θ*) scans in XRD and the in-plane data in 2D GIWAXS, respectively. As the PDMS ratio increased, the lamellar distance increased monotonically from 20.9 Å for Si0 to 38.4 Å for Si100. By contrast, the π-stacking distance decreased from 4.0 Å for Si0 to 3.5 Å for Si100. The increased lamellar distance with the higher PDMS content could be ascribed to the greater volume of the PDMS side chain compared with the branched alkyl side chain. The shorter π-stacking distance indicates that the π-planes were closer to each other with the PDMS side chains than with the branched alkyl side chains. This result coincides with the stronger interchain interaction observed in the UV–vis spectra and may reflect the smaller steric hindrance with the PDMS side chains. This could be advantageous for intermolecular charge transport in OFETs.

**Table 2.** Summary of *d*-spacings for the diffraction peaks observed in the XRD and GIWAXS patterns. GIWAXS, grazing-incidence wide-angle X-ray scattering; XRD, X-ray diffraction.

polymer	Si0	Si30	Si50	Si70	Si100
lamellar (100) (Å)^a^	20.9	29.1	34.0	35.9	38.4
π-stacking (001) (Å)^b^	4.0	3.6	3.6	3.6	3.5

^a^Distances obtained from the out-of-plane (*θ*/2*θ*) scans with the laboratory XRD.

^b^Distances obtained from the in-plane data in 2D GIWAXS.

To investigate the effect of a shorter π-stacking distance, we fabricated OFET devices with bottom-gate/top-contact configurations. A thin layer of the amorphous fluoropolymer CYTOP was used to passivate the SiO_2_ surface. Initially, the ordinary spin coating of the polymer solution onto the dielectric layer was adopted for the device preparation. However, we observed poor film quality and large hysteresis in the OFET measurements. To avoid these issues, we used a contact film transfer method, in which the polymer films were spin-coated onto the poly(styrene sulfonate) (PSS)/glass substrate and then transferred onto the silicon substrate with the CYTOP layer (see Material and methods for details) [30]. This method allows the use of the film surface for the charge transport channel with the bottom-gate configuration, which can lead to better performance and stability [31–33]. The typical OFET characteristics are presented in the electronic supplementary material, figure S4, and the hole mobilities of the copolymers in the saturated region are summarized in table 3. The mobilities decreased from 0.60 cm^2^ V^−1 ^s^−1^ for Si0 to 0.21–0.24 cm^2^ V^−1 ^s^−1^ for Si30 and Si50 as the proportion of PDMS side chains was increased to 50%. Further increasing the PDMS content in the copolymers decreased the mobility down to 0.0015 cm^2^ V^−1^ s^−1^ for Si100. The much lower mobility observed for Si100 may be partly attributed to its low molecular weight compared with the other copolymers. This result shows that the introduction of the PDMS side chain did not lead to an improvement in the mobility, in contrast to the results observed with other alkyl-based solubilizing units [16].

**Table 3.** Hole mobilities of the copolymers.

polymer	Si0	Si30	Si50	Si70	Si100
hole mobility (cm^2^ V^−1^ s^−1^)	0.60 ± 0.21	0.21 ± 0.09	0.24 ± 0.05	0.13 ± 0.03	0.0015

**Figure 4.**
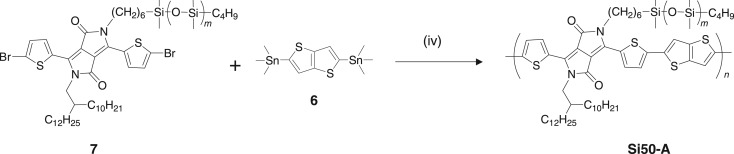
Synthetic route to the polymer Si50-A, with PDMS substitution on only one side of the main chain.

OFET performance is known to be sensitive to the molecular orientation in the transport channels that exist in a layer of several nanometres close to the dielectric interface. As we used the transferred polymer films to fabricate the OFETs, the surface structure of the films could be important for the performance. The random incorporation of PDMS side chains may affect the main-chain conformation, especially at the film surfaces. To further investigate the effect of the introduction of PDMS, we synthesized a DPP monomer unit asymmetrically substituted with PDMS and branched alkyl side chains (**7**) in a similar manner to the monomer **4** (see the electronic supplementary material for details) and used it in the copolymerization reaction with the thienothiophene monomer **6** (figure 4). The resulting polymer, referred to here as Si50-A, contains the same fraction of PDMS as Si50, but each DPP unit contains a PDMS side chain on only one side. This could alter the surface structure and packing characteristics in the thin films. It should be noted that the polymer is regiorandom in terms of the direction of the PDMS side chain.

The *M*_n_ and PDI of Si50-A were 87 kg mol^−1^ and 2.7, respectively. These values were considerably lower than those of Si50. The solubility in CHCl_3_ was 31 mg ml^−1^, which is higher than that for Si50. This may be the result of the different substitution patterns, although the lower molecular weight may also play a role. Si50 and Si50-A exhibited similar UV–vis absorption spectra in both CHCl_3_ solutions and thin films (see electronic supplementary material, figure S2). The XRD patterns of thin films of Si50 and Si50-A were also compared (see electronic supplementary material, figure S3). The Si50-A film possessed a lamellar distance of 34 Å and a π-stacking distance of 3.6 Å, which are similar to the corresponding values for the Si50 film. OFET devices using Si50-A were fabricated in the same way. The mobility of OFETs based on Si50-A was 0.22 ± 0.07 cm^2^ V^−1^ s^−1^, which is comparable with the devices based on Si50. Therefore, the different PDMS substitution patterns in Si50 and Si50-A seem to have little effect on the structures in thin films and the charge mobility in OFET devices.

Other than the molecular orientation at the film surface, there are still possible reasons for the observed deterioration of the mobility by the introduction of the PDMS side chains. A plausible reason is lower order of the π-stacking in the copolymers, even though the distance of the stacking became shorter. In addition, the flexibility of the PDMS with a low glass transition temperature may cause the thermal fluctuation of the structure, which could be detrimental to the charge transport. Further investigations are necessary to conclude the effects of the PDMS side chains on the charge transport.

## 3. Conclusion

We synthesized DPP-based copolymers to investigate the potential of PDMS as a solubilizing group in semiconducting polymers. The introduction of PDMS side chains greatly increased the solubility of the polymers, and the *d*-spacings of the π-stacking became shorter than those of polymers containing branched alkyl side chains in thin films. Although the charge mobility of the OFETs was not improved in this study by using the PDMS-containing polymers, the strategy of using PDMS as the solubilizing group could be useful for much larger π-conjugated polymer systems that have more severe problems in terms of solubility and processability.

## 4. Material and methods

### 4.1. Characterization

The molecular weights and PDI values were determined by gel permeation chromatography on a Shimadzu HPLC-8020 using a calibration curve based on polystyrene standards in CHCl_3_ at 40°C. ^1^H-NMR spectra were recorded on JEOL JNM-LA 300 or 400 MHz instruments using CDCl_3_ or DMSO-*d*_6_ as the solvent. MALDI-TOF-MS spectra were recorded on a Bruker Daltonics Ultraflex using dithranol as the matrix (10 mg ml^−1^ in CHCl_3_). UV–vis spectra were recorded on a JASCO V-650 spectrophotometer. XRD was performed by Rigaku Smart Lab. The incident angle for the in-plane measurements was 0.2°. 2D GIWAXS was measured on the BL19B2 and BL46XU beamlines at SPring-8 with an incident angle of 0.12°. The film samples were prepared by spin coating the polymer solution in CHCl_3_ on the Si/SiO_2_ (300 nm) substrate at 2500 r.p.m. for 60 s. The concentrations were 7 mg ml^−1^, 9 mg ml^−1^, 11 mg ml^−1^, 12 mg ml^−1^ and 15 mg ml^−1^ for Si0, Si30, Si50, Si70 and Si100, respectively. Photoelectron spectroscopy in air was performed on a Riken Keiki AC-2 instrument. The solubility of the polymers was tested by weighing out approximately 10 mg of the samples into a vial and adding CHCl_3_ by 50 µl incrementally until the complete dissolution of the polymer was observed by the naked eye.

### 4.2. Fabrication of field-effect transistors

The transistors were constructed on highly doped *n*-type Si substrates with 300-nm-thick SiO_2_. A CYTOP solution was spin-coated onto the SiO_2_ surface for surface passivation, with a thickness of 7–12 nm as determined by X-ray reflectivity measurements. The semiconducting polymer layer was transferred onto the SiO_2_ surface by a contact film transfer method [30]. In this method, PSS sodium salt and the polymer layers were successively spin-coated onto a glass substrate to form a multilayered film. This film was brought into contact with the silicon substrate upside down. One drop of water was placed on the edge of the two stacked substrates and allowed to selectively dissolve the PSS layer. Finally, the glass substrate was removed and the polymer film was transferred onto the silicon substrate. To complete the OFET fabrication, gold electrodes were evaporated onto the surface through a metal mask. The channel lengths were 50, 100, 150 and 200 µm and the width was 1000 µm. The average charge mobility was calculated from the values with all the channel lengths in the saturated region. The capacitance of the gate dielectric *C*_i_ was calculated from the ideal capacitance of SiO_2_ and CYTOP using the synthetic capacitance equation.

### 4.3. Synthesis

#### 4.3.1. 3,6-Dithiophene-2-yl-2,5-dihydropyrrolo[3,4-*c*]pyrrole-1,4-dione (**1**)

Na (10.62 g, 462 mmol) and FeCl_3_ (3.5 mg) were dissolved in *t*-amyl alcohol (240 ml), and the resulting mixture was stirred at 90°C until the complete dissolution of Na [34]. After cooling to 60°C, thiophene-2-carbonitrile (25 g, 231 mmol) was added and the mixture was again heated to 90°C. A solution of dibutyl succinate (18.88 ml, 92.4 mmol) in *t*-amyl alcohol (70 ml) was added dropwise over 3 h, and the temperature was maintained at 90°C for 20 h. After cooling to 50°C, AcOH was added and the mixture was then refluxed at 130°C for 10 min. After filtration, the residue was washed with water and hot MeOH. The solid was dried under vacuum. Yield: 17.1 g (62%). ^1^H-NMR (DMSO-*d*_6_, 300 MHz): *δ* (ppm) 11.20 (s, 2H), 8.22 (dd, *J* = 5.1, 1.1 Hz, 2H), 7.93 (dd, *J* = 5.1, 1.1 Hz, 2H), 7.28 (dd, *J* = 4.9, 3.8 Hz, 2H).

#### 4.3.2. 3,6-Bis-(5-bromothiophene-2-yl)-2,5-dihydropyrrolo[3,4-c]pyrrole-1,4-dione (**2**)

*N*-Bromosuccinimide (NBS, 4.41 g, 24.8 mmol) was slowly added to a solution of **1** (3.42 g, 11.4 mmol) in CHCl_3_ (1 l) over a period of 15 min. The solution was then stirred at room temperature for 48 h in the dark. The reaction mixture was poured into MeOH (6 l), filtered and dried under vacuum at 40°C overnight. Yield: 3.64 g (70%). ^1^H-NMR (DMSO-*d*_6_, 300 MHz): *δ* (ppm) 11.35 (s, 2H), 7.95 (d, *J* = 4.3 Hz, 2H), 7.46 (d, *J* = 4.3 Hz, 2H).

#### 4.3.3. 3,6-Bis-(5-bromothiophene-2-yl)-2,5-di(hex-5-en-1-yl)pyrrolo[3,4-*c*]pyrrole-1,4-dione (**3**)

To a suspension of **2** (0.425 g, 0.928 mmol) and potassium carbonate (0.534 g, 3.86 mmol) in anhydrous DMF (9.5 ml), 6-bromo-1-hexene (0.45 ml, 2.27 mmol) was injected through a septum under N_2_ [16]. The mixture was stirred for 15 h at 100°C and then poured into water (100 ml). The reaction solution was filtered and washed with CHCl_3_ to remove the starting material with poor solubility. After removal of the solvent under reduced pressure, the deep purple solid was collected and purified by silica gel chromatography (CHCl_3 _: Hex = 1 : 1). Yield: 0.128 g (22%). ^1^H-NMR (300 MHz, CDCl_3_): *δ* (ppm) 8.67 (d, *J* = 4.0 Hz, 2H), 7.24 (d, *J* = 4.0 Hz, 2H), 5.73–5.86 (m, 2H), 4.95–5.05 (m, 4H), 4.00 (t, *J* = 7.7 Hz, 4H), 2.12 (q, *J* = 7.0 Hz, 4H), 1.69–1.79 (m, 4H), 1.46–1.54 (m, 4H). MALDI-TOF-MS: *m/z* 619.92 (*z*^+^), calcd.: 619.98.

#### 4.3.4. 3,6-Bis-(5-bromothiophene-2-yl)-2,5-bis(6-(polydimethylsiloxanyl)hexyl)pyrrolo[3,4-*c*]pyrrole-1,4-dione (**4**)

To a solution of **3** (200 mg, 0.321 mmol) in anhydrous toluene (5 ml) under N_2_, monohydride-terminated PDMS (Gelest, *M*_n_: 800–900, 1.4 ml, 1.28 mmol) was injected through a septum, followed by the addition of a drop of Karstedt's catalyst (platinum-divinyltetramethyldisiloxane complex in xylene, 3 wt%) [16]. The resulting mixture was stirred at 50°C under N_2_ for 48 h and then filtered. After removal of the solvent under reduced pressure, the viscous purple liquid was purified by silica gel chromatography (CH_2_Cl_2 _: Hex = 1 : 1). Yield: 377 mg (50%). ^1^H-NMR (400 MHz, CDCl_3_): *δ* (ppm) 8.68 (d, *J* = 4.0 Hz, 2H), 7.24 (d, *J* = 4.0 Hz, 2H), 3.98 (t, *J* = 7.9 Hz, 4H), 1.70 (m, 4H), 1.27–1.53 (m, 20H), 0.88 (t, 6.6 Hz, 10 H), 0.51–0.55 (m, 10 H), 0.01–0.03 (m, 200 H).

#### 4.3.5. General procedure for the synthesis of the copolymers (Si30, Si50, Si70 and Si100)

Monomer **4**, 3,6-bis(5-bromo-2-thienyl)-2,5-dihydro-2,5-di(2′-decyltetradecyl)pyrrolo[3,4-*c*]pyrrolo-1,4-dione (**5**, SunaTech, Inc., sum of **4** and **5**: 0.2 mmol) and 2,5-bis(trimethylstannyl)thieno[3,2-*b*]thiophene (**6**, Sigma-Aldrich, 0.2 mmol) were placed in a microwave reaction tube and dissolved in degassed toluene (5 ml). After adding Pd_2_(dba)_3_·CHCl_3_ (6.65 mg) and P(*o*-tolyl)_3_ (3.33 mg), the tube was sealed, placed in a microwave reactor (Biotage Initiator+) and heated at 135°C for 30 min with stirring. The reaction mixture was poured into MeOH and the precipitate was collected by filtration. The deep blue solid was washed successively with acetone, hexane and CH_2_Cl_2_ in a Soxhlet extractor. The remaining solid was then extracted with CHCl_3_, precipitated with acetone, collected by filtration through a 0.2-μm membrane filter and dried under vacuum overnight. Yields: 45% (Si30), 62% (Si50), 54% (Si70) and 78% (Si100).

#### 4.3.6. Synthesis of DT-PDPP2T-TT (Si0)

Si0 was synthesized in a similar manner to the copolymers using **5** (200 mg, 0.176 mmol) and **6** (81.9 mg, 0.176 mmol) in a mixture of toluene (8 ml) and DMF (0.8 ml) with Pd_2_(dba)_3_·CHCl_3_ (4.8 mg) and P(*o*-tolyl)_3_ (2.4 mg) as the catalyst system [21]. The reaction was conducted at 115°C for 48 h. The polymer was purified by the same procedure as for the copolymers. Yield: 160 mg (82%).

## Supplementary Material

Electric Supplementary File

## Data accessibility

Electronic supplementary material is available. For detailed characterizations, see dryad: http://dx.doi.org/10.5061/dryad.3rk3h. [35].
