# Supplementary material for: Synthesis of diketopyrrolopyrrole-based polymers with polydimethylsiloxane side chains and their application in organic field-effect transistors
Source: R Soc Open Sci. 2018 Mar 14;5(3):172025. doi: 10.1098/rsos.172025 (PMC5882725; doi:10.1098/rsos.172025)
Supplement: Electric Supplementary File [file rsos172025supp1.pdf]

Supporting Information for

Synthesis of diketopyrrolopyrrole-based polymers with polydimethylsiloxane side chains and their application in organic field-effect transistors

Inori Ohnishi[1,2], Kazuhito Hashimoto[2], and Keisuke Tajima[1]*

[1]RIKEN Center for Emergent Matter Science (CEMS), 2-1 Hirosawa, Wako 351-0198, Japan

[2]Department of Applied Chemistry Graduate School of Engineering, The University of Tokyo, 7-3-1 Hongo, Bunkyo-ku, Tokyo 113-8656, Japan

Synthesis of asymmetrically substituted DPP monomer (7)

11-(Bromomethyl)tricosane (**8**)[1]

Br_2 (6.44 mL, 0.125 mol, 1.05 eq.) was added to a solution of triphenylphosphine (32.8 g, 0.125 mol, 1.05 eq.) in anhydrous CH_2Cl_2 (110 mL) at 0 °C to form a pale yellow slurry. A solution of 2-decyl-1-tetradecanol (50 mL, 0.12 mol, 1.0 eq.) in CH_2Cl_2 was added dropwise at 0 °C via an addition funnel. The resulting mixture was stirred at room temperature overnight to afford an orange solution. The organic solvent was then evaporated under reduced pressure. After quenching with sodium thiosulfate solution, the organic phase was extracted with hexane, dried over $MgSO_4$, and filtered. The filtrate was concentrated under reduced pressure. The solid residue was extracted three times with hexane. After filtration, the resulting orange oil was purified by silica gel column chromatography (silica gel, hexane) to afford **8** as a colorless oil. Yield: 35.6 g (73%). ^{1}H-NMR (300 MHz, $CDCl_3$): δ (ppm) 3.42 (d, *J* = 4.4 Hz, 2H), 1.58 (m, 1H), 1.42–1.08 (m, 40H), 0.88 (t, *J* = 6.4 Hz, 6H).

3,6-Dithiophene-2-yl-2-(hex-5-en-1-yl)-5-hydropyrrolo[3,4-*c*]pyrrole-1,4-dione (**9**)[3]

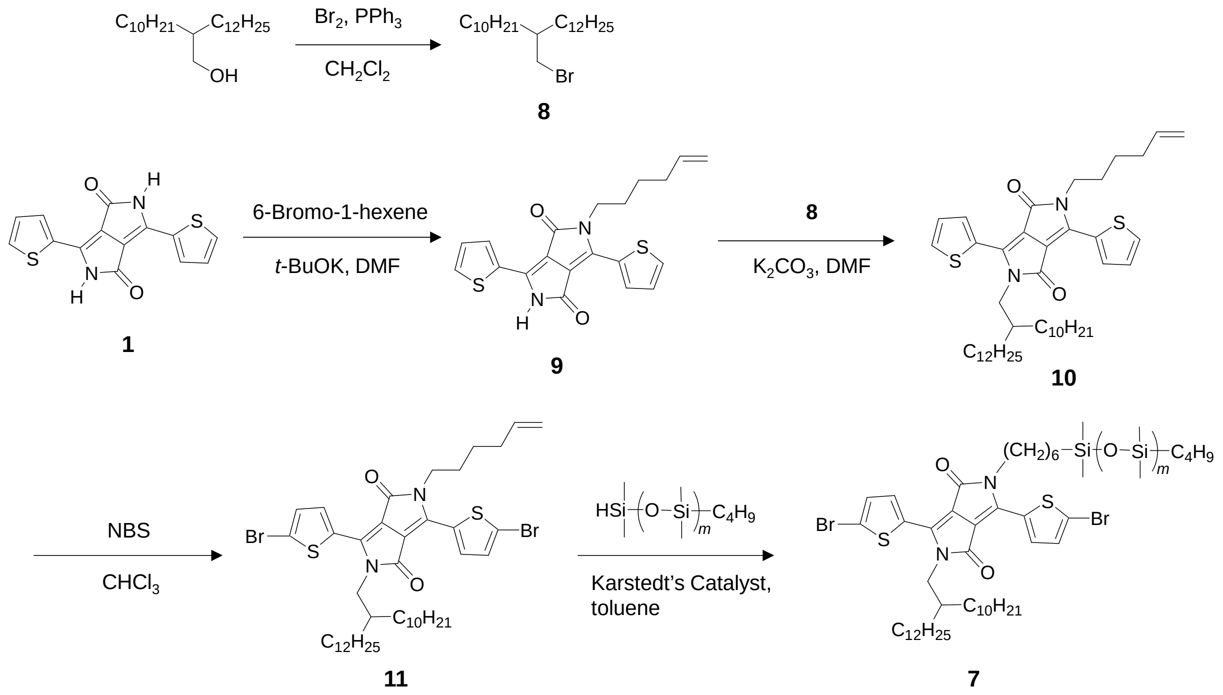

Fig. S1 Synthetic scheme for the asymmetrically substituted DPP monomer **7**.

Compound **1** (8.64 g, 28.77 mmol) was dispersed in a 1 L round-bottom flask containing anhydrous DMF (350 mL). The suspension was purged with N_2 for 20 min. *t*-BuOK (8.16 g, 72.21 mmol) was then added, causing an immediate color change from red to blue, and the resulting mixture was stirred for 20 min at room temperature. 6-Bromo-1-hexene (5.50 mL, 41.95 mmol) was then added dropwise under nitrogen. The mixture was stirred for 3 h at room temperature and then poured into water (800 mL) and stirred for an additional 1 h. The reaction solution was filtered and extracted with $CHCl_3$. The purple solid (6.95 g) obtained after removal of the solvent was then directly used in the next reaction without further purification.

3,6-Dithiophene-2-yl-2-(hex-5-en-1-yl)-5-(decyltetradecyl)pyrrolo[3,4-*c*]pyrrole-1,4-dione (**10**)[4]

To a suspension of **9** (6.55 g) and *t*-BuOK (4.08 g, 36.34 mmol) in anhydrous DMF (155 mL), **8** (15.17 g, 36.34 mmol) was injected through a septum under N_2. The mixture was stirred for 15 h at 100 °C and then poured into water (1 L). The reaction mixture was then extracted with $CHCl_3$ and dried over $MgSO_4$.

After removal of the solvent under reduced pressure, the starting material was completely removed by precipitation in hexane and the residue was purified by silica gel column chromatography (first with $CHCl_3$ and then with EtOAc:hexane [4:96]). The product was further purified by slow precipitation in MeOH and recrystallization in methanol:$CHCl_3$ (1:1). Yield: 2.66 g (13% from **1**). ^{1}H-NMR (400 MHz, $CDCl_3$): δ (ppm) 8.89 (ddd, *J* = 42.0, 3.9, 1.0 Hz, 2H), 7.52-7.64 (m, 2H), 7.26–7.30 (m, 2H), 5.75–5.85 (m, 1H), 4.94–5.03 (m, 2H), 4.09 (t, *J* = 7.7 Hz, 2H), 4.01 (d, *J* = 7.7 Hz, 2H), 2.12 (q, *J* = 7.2 Hz, 2H), 1.89 (m, 1H), 1.74–1.82 (m, 2H), 1.49–1.54 (m, 2H), 1.21–1.29 (m, 40H), 0.82-0.89 (m, 6H). MALDI-TOF-MS: *m*/*z* 718.32 (z^+), calcd.: 718.46.

3,6-Bis-(5-bromothiophene-2-yl-2-(hex-5-en-1-yl)-5-(decyltetradecyl)pyrrolo[3,4-*c*]pyrrole-1,4-dione (**11**)

NBS (1.31 g, 7.34 mmol) was added dropwise to a solution of compound **10** (2.65 g, 3.62 mmol) in $CHCl_3$ (200 mL) at 0 °C. The solution was protected from light and stirred for 48 h. The reaction mixture was poured into water (200 mL) and extracted three times with $CHCl_3$. The organic layer was dried over $MgSO_4$ and the solvent was evaporated under reduced pressure. The crude product was purified by silica gel column chromatography (EtOAc:hexane [2:98]). Yield: 957 mg (30%). ^{1}H-NMR (400 MHz, $CDCl_3$): δ (ppm) 8.58 (dd, *J* = 38.6, 4.3 Hz, 2H), 7.23–7.21 (m, 2H), 5.80 (m, 1H), 5.00 (m, 2H), 4.00 (t, *J* = 7.1 Hz, 2H), 3.92 (d, *J* = 8.2 Hz, 2H), 2.12 (q, *J* = 7.1 Hz, 2H), 1.78 (m, 1H), 1.68 (m, 2H), 1.42–1.50 (m, 2H), 1.20–1.26 (m, 40H), 0.87 (t, 6H). MALDI-TOF-MS: *m*/*z* 874.12 (z^+), calcd.: 874.28.

3,6-Bis-(5-bromothiophene-2-yl-2-(6-(polydimethylsiloxanyl)hexyl)-5-(decyltetradecyl)pyrrolo[3,4-*c*]pyrrole-1,4-dione (**7**)[4]

To **11** (670 mg, 0.810 mmol) in anhydrous toluene (5.8 mL) under N_2 atmosphere, monohydride-terminated polydimethylsiloxane (Gelest, M_n: 800–900, 0.81 mL, 0.972 mmol) was injected through a septum, followed by the addition of a drop of Karstedt's catalyst (platinum-divinyltetramethyldisiloxane complex in xylene, 3 wt %). The resulting mixture was stirred at 50 °C for 48 h under N_2 and then filtered.

After removal of the solvent under reduced pressure, the resulting viscous purple liquid was purified by silica gel chromatography (EtOAc:hexane [2:98]). Yield: 829 mg (65%). ^{1}H-NMR (400 MHz, $CDCl_3$): δ (ppm) 8.65 (dd, *J* = 39.7, 4.3 Hz, 2H), 7.21-7.29 (m, 2H), 3.98 (t, *J* = 7.6 Hz, 2H), 3.92 (d, *J* = 7.9 Hz, 2H), 1.87 (m, 1H), 1.70 (m, 2H), 1.22-1.38 (m, 70H), 0.87 (m, 10H), 0.51-0.55 (m, 6H), 0.04-0.15 (m, 90H).

Synthesis of Si50-A

Compound **7** (353 mg, 0.208 mmol), **6** (96.89 mg, 0.208 mmol), and degassed toluene (5 mL) were added to a microwave reaction tube. After the addition of $Pd_2(dba)_3 \cdot CHCl_3$ (6.61 mg) and P(*o*-tolyl)$_3$ (3.38 mg), the tube was sealed and placed into a microwave reactor. The reaction was performed under microwave irradiation for 30 min at a reaction temperature of 135 °C. The product was precipitated in MeOH and collected by filtration. The deep blue solid was then washed successively with acetone, hexane, and CH_2Cl_2 in a Soxhlet extractor. The remaining solid was then extracted with $CHCl_3$, precipitated with acetone, collected by filtration through a 0.2-μm membrane filter, and dried under vacuum overnight. Yield: 213 mg (60%).

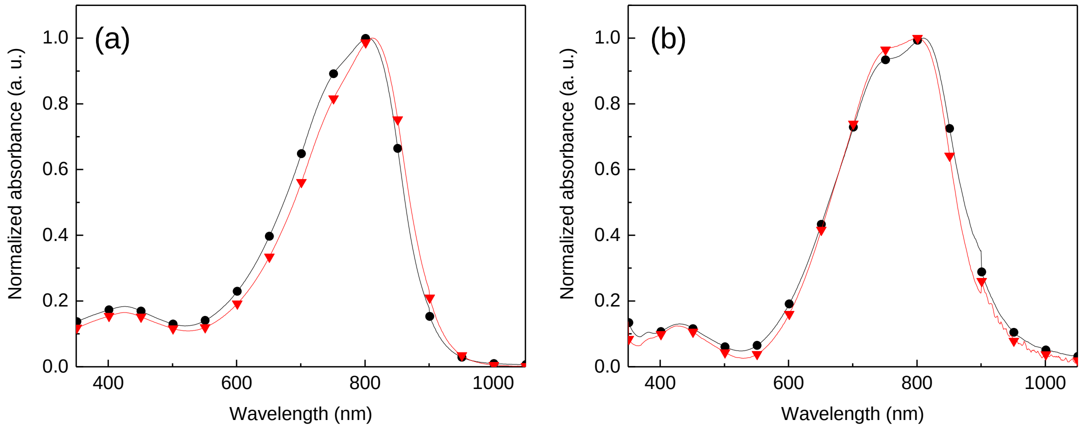

Fig. S2 Normalized UV–vis absorption spectra of Si50 (red curves) and Si50-A (black curves) in (a) $CHCl_3$ solutions and (b) thin films.

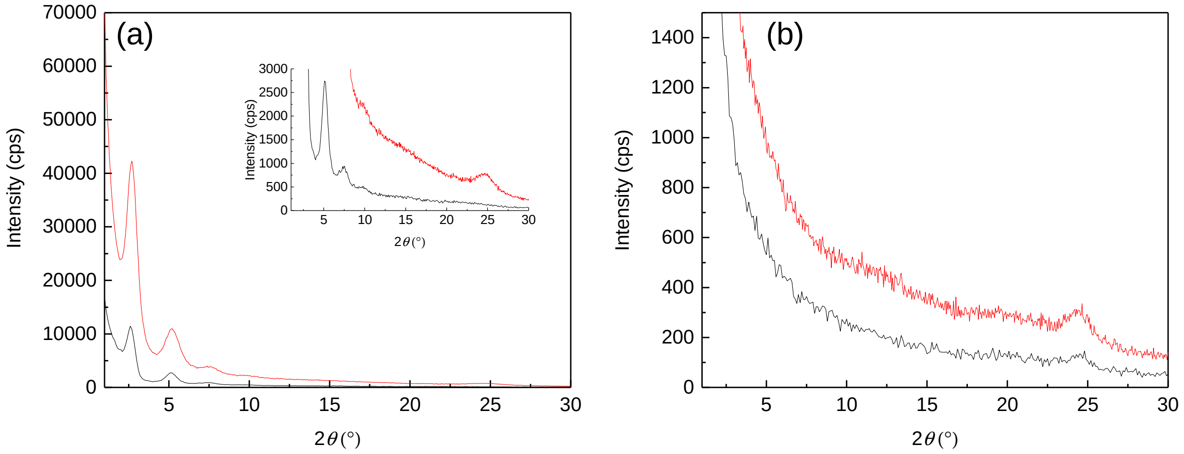

Fig. S3 (a) Out-of-plane and (b) in-plane XRD patterns of films of Si50 (red curves) and Si50-A (black curves). The inset in (a) is an expansion of the same graph. The Si50 and Si50-A films were annealed at 250 °C and 300 °C, respectively, for 5 min.

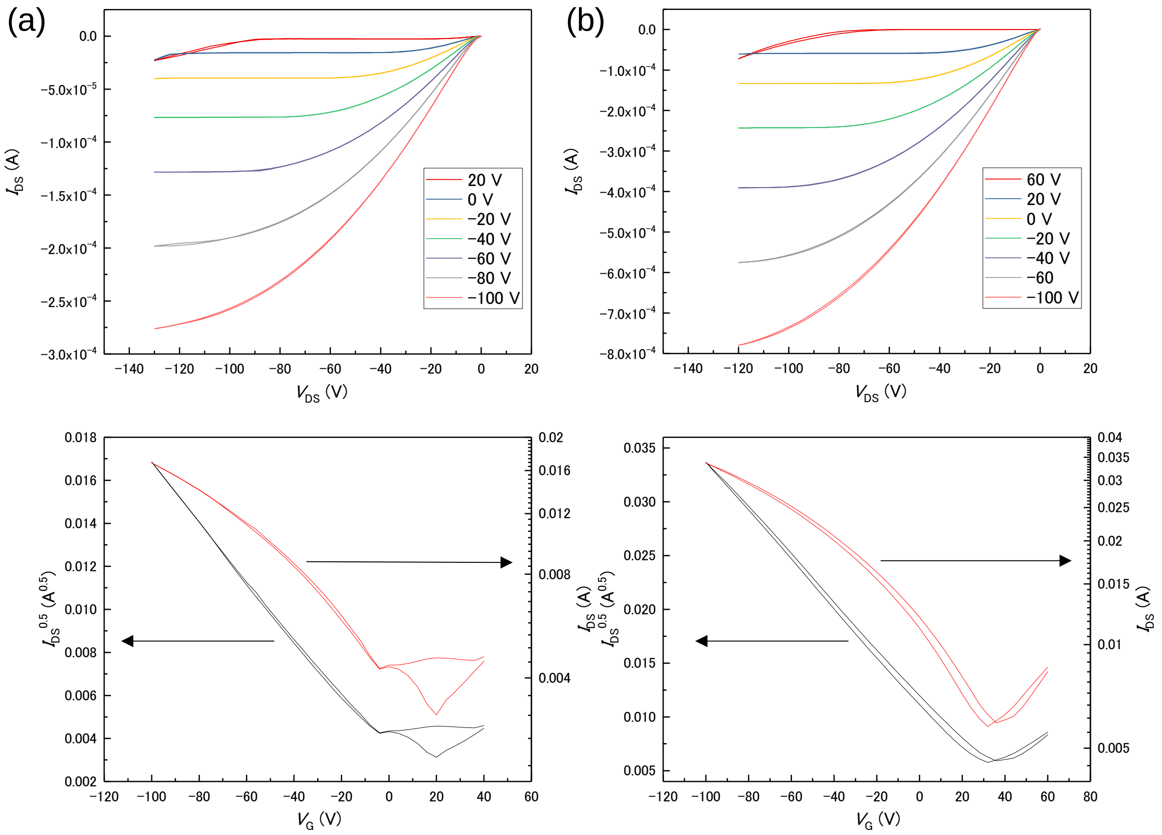

Fig. S4 Typical output and transfer curves of OFET devices based on (a) Si0 and (b) Si30 films. The channel length and width were 200 μm and 1000 μm, respectively. The copolymer of DPP and thienothiophene was reported to be an ambipolar material[2] and the transistor does not shut off completely even at V_G of around 0 V and above because of the electron injection.

References

1. B. Fu, J. Baltazar, A. R. Sankar, P.-H. Chu, S. Zhang, D. M. Collard and E. Reichmanis, *Adv. Funct. Mater.*, 2014, **24**, 3734-3744.
2. W. Li, K. H. Hendriks, W. S. Roelofs, Y. Kim, M. M. Wienk and R. A. Janssen, *Adv. Mater.*, 2013, **25**, 3182-3186.
3. M. V. Raju and H. C. Lin, *Org. Lett.*, 2013, **15**, 1274-1277.

4. J. Mei, D. H. Kim, A. L. Ayzner, M. F. Toney and Z. Bao, *J. Am. Chem. Soc.*, 2011, **133**, 20130-20133.
